# Implementation Determinants and System-Level Barriers in Supplementary Feeding Programs for Children Under Five in Low- and Middle-Income Countries: A Critical Narrative Review

**DOI:** 10.3390/children13101392

**Published:** 2026-10-13

**Authors:** Mohamed Anas Patni, Sonali G. Choudhari, Biji Thomas George, Rasha Aziz Attia Salama, Shadha Nasser Bahutair, Mushirabanu Sharifmiyan Akikwala

**Affiliations:** 1 Department of Community Medicine, RAK College of Medical Sciences, RAK Medical and Health Sciences University, Ras Al Khaimah 11172, United Arab Emirates; rasha.aziz@rakmhsu.ac.ae; 2 Department of Community Medicine, School of Epidemiology and Public Health, Jawaharlal Nehru Medical College, Adani Institute of Higher Education and Research, Wardha 442107, India; sonali.choudhari@dmiher.edu.in; 3 Department of General Surgery, RAK College of Medical Sciences, RAK Medical and Health Sciences University, Ras Al Khaimah 11172, United Arab Emirates; biji@rakmhsu.ac.ae; 4 Department of Community Medicine, Kasr El Aini Faculty of Medicine, Cairo University, Cairo 11956, Egypt; 5 Department of Obstetrics & Gynecology, RAK College of Medical Sciences, RAK Medical and Health Sciences University, Ras Al Khaimah 11172, United Arab Emirates; shadha@rakmhsu.ac.ae; 6 Department of Pediatrics, Shifa Al Jazeera Medical Centre, Ras Al Khaimah 11172, United Arab Emirates; drmushira87@gmail.com

**Keywords:** supplementary feeding, child undernutrition, implementation science, health systems, program sustainability, community health workers, equity, low- and middle-income countries

## Abstract

**Highlights:**

**What are the main findings?**

- Supplementary feeding programs for children under five in low- and middle-income countries are constrained by interacting financing, supply-chain, workforce, monitoring and household-level barriers.
- These barriers disrupt the delivery pathway from program design and procurement to household uptake and child consumption, reducing coverage, continuity, adherence and nutritional benefit.

**What are the implications of the main findings?**

- Effective programs require coordinated investment in sustainable financing, reliable supply systems, supported frontline workers and usable monitoring data—not food provision alone.
- Community engagement, culturally acceptable foods, local ownership and equity-sensitive implementation are essential for sustained programs uptake and nutritional impact.

**Abstract:**

**Background**: Supplementary feeding programs are widely used to prevent or manage child undernutrition, but their population-level benefit depends on implementation. Objective: To critically synthesize multilevel determinants of program reach, continuity, utilization, equity and sustainability in low- and middle-income countries (LMICs). **Methods:** We conducted a critical narrative review of peer-reviewed and grey literature identified through iterative database searching and targeted verification. Sources were charted by setting, design, program, implementation determinant, consequence, evidence directness, limitation and narrative credibility. Direct, related and indirect evidence were weighted differently in the synthesis. **Results**: Direct and related evidence most consistently linked program performance to financing and supply continuity, workforce preparation and supervision, food acceptability, caregiver understanding, equitable access and monitoring linked to corrective action. Direct evidence was concentrated in South Asia and selected programs in Indonesia, Iran and Ethiopia. Evidence concerning digital delivery, father-inclusive approaches, community-health-worker incentives and donor transition was largely indirect and supports hypotheses rather than established program effects. **Conclusions**: Reliable delivery systems and effective child-level utilization are necessary for supplementary feeding programs to contribute to nutritional outcomes. Recommendations supported mainly by indirect evidence require prospective evaluation in supplementary feeding settings, particularly in underrepresented LMIC regions.

## 1. Introduction

Child nutrition poverty is increasingly described through the concept of child food poverty, defined as the inability of young children to access and consume a nutritious and diverse diet. UNICEF estimated that 181 million children under five, approximately one in four globally, were living in severe child food poverty in 2024. This deprivation threatens survival, physical growth, cognitive development and the ability to reach developmental potential [1].

Nutrition is also one dimension of the broader construct of child well-being, which includes material living conditions, health-care access, caregiving, learning, protection, social relationships and opportunities for development. Leriou’s multidimensional study in Attica placed nutrition alongside home conditions, guardian employment, health care, moral education and leisure, and found nutrition to be an important contributor to measured child well-being [2]. Although that study involved school-age children in a high-income setting and is not direct evidence for under-five programs in LMICs, it usefully demonstrates why nutritional outcomes should be interpreted within children’s wider social and developmental circumstances.

Recent LMIC evidence similarly shows that economic gains do not automatically translate into improved child nutrition. Among smallholder farm households in Nigeria, higher agricultural commercialization did not consistently correspond to better anthropometric status among children under five; child age, access to health care, infrastructure and household characteristics also mattered [3]. Recent commentary on child food poverty likewise emphasizes diet quality and structural inequities rather than hunger or household income alone [4]. Together, this literature supports responses that combine nutritious foods with caregiver support, accessible health services, social protection, healthier food environments and systems for identifying and monitoring vulnerable children [1,3,4].

Supplementary feeding programs sit within this broader child well-being response. They may reduce dietary gaps and provide an entry point for counselling, growth monitoring, referral and connection to health or social services. However, they cannot by themselves resolve poverty, unaffordable healthy diets, infection, unsafe environments or weak services. Their contribution to child well-being therefore depends on equitable reach, continuity, acceptability and actual consumption by the intended child, and broader well-being effects should not be assumed without direct measurement.

Within this broader context, child undernutrition remains a major public-health challenge in low- and middle-income countries (LMICs). Wasting, stunting and underweight arise through interacting pathways that include inadequate dietary intake, infection, food insecurity, poverty, unsafe environments and limited access to responsive health and nutrition services [5,6]. Supplementary feeding is therefore best understood as one component of a wider system of prevention, early identification, treatment and social support rather than as a stand-alone commodity intervention.

The term supplementary feeding encompasses several distinct approaches. Preventive or blanket programs provide additional foods to defined at-risk populations, whereas targeted supplementary feeding is generally intended for children with moderate wasting or other specified nutritional vulnerability. Broader supplementary nutrition programs may distribute take-home rations, cooked meals, fortified blended foods or locally prepared foods through community and social-protection platforms. Ready-to-use supplementary foods are distinct from ready-to-use therapeutic foods, which are used principally in the management of severe wasting. Therapeutic feeding and community-based management of acute malnutrition were included in this review only when they provided transferable evidence about procurement, referral, workforce, monitoring, integration or sustainability [5,7,8,9]. For infants younger than six months, breastfeeding assessment and specialized clinical support remain central; food supplementation for this group should not be inferred from evidence concerning children aged 6–59 months [5].

Evidence from complementary feeding trials and syntheses suggests that food support combined with caregiver counselling can improve dietary intake and may yield modest growth benefits, although effects vary by age, baseline nutritional status, intervention intensity and context [9,10]. Implementation quality is one explanation for this variation. Irregular supplies, poor storage, limited workforce preparation, weak caregiver engagement and fragmented monitoring can reduce reach, continuity and effective child intake even when the nutritional product is technically appropriate [7,11,12,13,14,15].

Implementation barriers operate at interacting levels. Household food insecurity and intra-household sharing may dilute the dose consumed by the target child; community engagement and geographic access influence participation; workforce workload and supervision shape service quality; and financing, procurement, information systems and governance determine whether services remain available at scale [11,12,13,14,15]. Existing evidence is dispersed across qualitative studies, process evaluations, trials, systematic reviews and operational reports, while some potentially useful mechanisms are reported only in adjacent maternal–child-health or community-health-worker programs.

This critical narrative review therefore synthesizes implementation determinants affecting the reach, continuity, utilization, equity and sustainability of supplementary feeding programs for children under five in LMICs. It considers these programs within the broader context of child food poverty and child well-being, while retaining a specific focus on program delivery. It distinguishes direct program evidence from related acute-malnutrition or child-nutrition evidence and from indirect evidence concerning transferable delivery mechanisms. It also examines major implementation trade-offs and identifies priorities for policy, practice and research.

## 2. Materials and Methods

### 2.1. Review Design and Reporting Approach

A critical narrative-review design was used because the question required integration of heterogeneous qualitative, quantitative, mixed-method, implementation, policy and program-evaluation evidence rather than estimation of a pooled intervention effect. The review was prepared with reference to SANRA and methodological guidance emphasizing objectives, transparent searching, critical interpretation and appropriate presentation of evidence [16,17]. PRISMA-S principles informed the description of information sources, but the review was not designed or reported as a systematic review and does not claim exhaustive retrieval [18].

### 2.2. Review Question and Conceptual Scope

The review addressed the following question: Which implementation determinants at policy, health-system, program, workforce, community, household and caregiver levels influence the reach, continuity, utilization, equity and sustainability of supplementary feeding programs for children under five in LMICs?

Eligible program types included preventive or blanket supplementary feeding, targeted supplementary feeding, take-home rations, cooked-meal services, fortified blended foods, locally produced supplementary foods, lipid-based nutrient supplements and ready-to-use supplementary foods. Evidence concerning therapeutic feeding or community-based management of acute malnutrition (CMAM) was treated as related rather than equivalent evidence. Complementary feeding counselling, breastfeeding promotion, digital health, community-health-worker and maternal–child-health interventions were treated as indirect unless they were embedded within a relevant nutrition program.

### 2.3. Information Sources and Search Approach

Studies were identified during manuscript development through iterative searches of PubMed/MEDLINE, Scopus, Web of Science Core Collection, Google Scholar and the Cochrane Library. Search development began with broad combinations of population, program and implementation terms and was refined using terminology encountered during title and abstract review. Backward citation checking and targeted searches for named programs were also used. Grey literature was sought from the World Health Organization, UNICEF, the World Food Programme, the World Bank, national nutrition programs and government websites. Core concepts included children under five; supplementary feeding, take-home rations, fortified blended foods, ready-to-use supplementary foods and CMAM; and implementation, acceptability, adherence, coverage, supply chains, workforce, monitoring, financing, equity, sustainability and scale-up. No publication-year restriction was applied, and English-language sources were used. Targeted contextual searches also combined terms for child food poverty, nutrition poverty, dietary deprivation and child well-being with terms for children under five and LMICs. These contextual sources were used to frame the problem and its wider determinants rather than to establish the effectiveness of supplementary feeding programs.

The initial discovery searches were iterative and were not prospectively archived at record level. Exact historical database strings, retrieval counts and a retrospective PRISMA flow diagram therefore cannot be reconstructed reliably. During final manuscript development, targeted verification of PubMed-indexed literature and WHO, UNICEF and World Food Programme sources was completed on 18 August 2026 using the reproducible core strategy reported in Supplementary Box S1. The implementation evidence set was closed on that date. A targeted contextual update addressing child food poverty and child well-being was completed on 29 September 2026 using the terms described above based on reviewers’ comments. Supplementary Box S1 distinguishes the documented verification strategy from the earlier exploratory searches, so the review should be interpreted as a structured critical synthesis rather than an exhaustive systematic review.

Because the original discovery searches and record-level selection decisions were not prospectively documented, the completeness of retrieval and the extent of selection bias cannot be assessed. The final evidence base may therefore reflect both the available literature and author judgment during source identification and selection.

### 2.4. Eligibility Criteria

Sources were eligible when they concerned children aged 0–59 months, their caregivers, frontline workers, managers or policymakers; addressed a supplementary feeding or implementation-relevant child-nutrition program in an LMIC; and reported barriers, facilitators, acceptability, adherence, participation, coverage, equity, targeting, workforce, supervision, supply, financing, governance, monitoring, integration, scale-up or sustainability. Quantitative, qualitative and mixed-method studies; trials with implementation findings; evidence synthesis; program evaluations; policy documents; and technical reports were considered.

Sources were excluded when they addressed biological or clinical efficacy without implementation relevance, were conducted exclusively in high-income countries, focused only on populations outside the under-five age group, or provided insufficient methodological or contextual information. English-language evidence was used. No publication-year restriction was imposed.

### 2.5. Source Selection and Charting

Sources were selected within a single iterative workflow rather than through duplicate independent systematic-review screening. Titles and abstracts were examined for population, program and implementation relevance; potentially eligible full texts were then assessed against the criteria in Section 2.4. Reports were excluded when they concerned an ineligible population or setting, lacked an implementation focus or provided insufficient methodological or contextual information. Duplicate citations were consolidated during charting, and no automated screening tool was used. Information was charted by citation, country or region, design, population, program type, implementation determinant, consequence, evidence directness and principal limitation. The source-level chart is provided in Supplementary Table S1. Because the initial searches were not archived, record-level screening counts and exclusion totals are not reported.

### 2.6. Evidence Directness and Credibility

Evidence directness was judged separately from methodological credibility. Direct evidence examined a supplementary feeding or supplementary nutrition program for children under five and could support program-specific conclusions. Related evidence concerned CMAM, therapeutic feeding, complementary feeding or broader child-nutrition services with closely aligned delivery mechanisms and was used as supporting evidence. Indirect evidence came from adjacent community-health-worker, maternal–child-health, digital-health or scale-up programs. It was used to explain plausible mechanisms and generate implementation hypotheses, not to establish effectiveness within supplementary feeding programs.

A formal design-specific risk-of-bias instrument was not applied. Narrative credibility judgments considered five features: clarity of the objective and setting; appropriateness of the design for the question; transparency of sampling, data collection and analysis; acknowledgement of limitations; and consistency between the reported evidence and the authors’ interpretation. Higher credibility was assigned to evidence-informed guidelines and evidence syntheses with clear search, selection and appraisal methods. Moderate credibility was assigned to peer-reviewed empirical or process-evaluation studies with methods sufficient for interpretation. Contextual credibility was assigned to protocols, broad narrative reviews, selected grey literature and sources with limited reporting or remote relevance. These author-developed categories are not a validated appraisal framework, are not equivalent to risk-of-bias ratings and cannot establish certainty of effect. The criteria and source-level judgments are reported in Supplementary Table S1. These author-developed categories were used solely as an interpretive aid for organizing and weighting heterogeneous evidence. They have not been formally validated and should not be interpreted as substitutes for design-specific critical appraisal, risk-of-bias assessment or certainty-of-evidence grading.

### 2.7. Narrative Synthesis

Initial deductive categories reflected the program-delivery pathway and health-system functions: program design and targeting; procurement, production, storage and distribution; financing; workforce training, workload, motivation and supervision; governance; information systems; community participation; caregiver adherence; food acceptability; equity; integration; and sustainability. Evidence was compared within and across themes to identify convergence, contradiction, context dependence and research gaps. Quantitative findings were reported as presented in the original sources and were not pooled or recalculated. Conclusions judged to have stronger support required convergent direct evidence or direct evidence supported by closely related nutrition evidence. Indirect or contextual evidence could refine an explanation or identify a strategy for testing but did not independently justify a program recommendation. Credibility judgments therefore influenced the weight given to a source, while directness determined how far its findings could be transferred to supplementary feeding.

### 2.8. Ethical Consideration

Ethical approval was not required because the review used published and publicly available sources and did not involve human participants or identifiable information.

## 3. Profile and Directness of the Evidence

The evidence base combined direct supplementary feeding evaluations with related acute malnutrition and child nutrition studies and a substantial body of indirect implementation evidence. Direct studies most often concerned local supplementary feeding, India’s Integrated Child Development Services and take-home rations, ready-to-use supplementary foods or national food-support programs. Related evidence was dominated by CMAM integration, complementary feeding, nutrition information systems and broader child nutrition services. Indirect studies were most frequently used to interpret workforce supervision, digital delivery, family engagement, donor transition and scale-up. Table 1 shows how each evidence category contributed to the main conclusions and the limits placed on interpretation.

Geographic coverage was uneven. Direct evidence was concentrated in South Asia and selected country programs in Indonesia, Iran and Ethiopia. Latin America, the Middle East outside Iran, fragile states and several African subregions were sparsely represented. The review therefore identifies mechanisms that recurred in the available evidence, but it cannot establish their prevalence, relative importance or preferred response across all LMICs. Policy implications require local assessment of food systems, delivery platforms, administrative capacity, conflict exposure, geography and household vulnerability.

**Figure 1:**
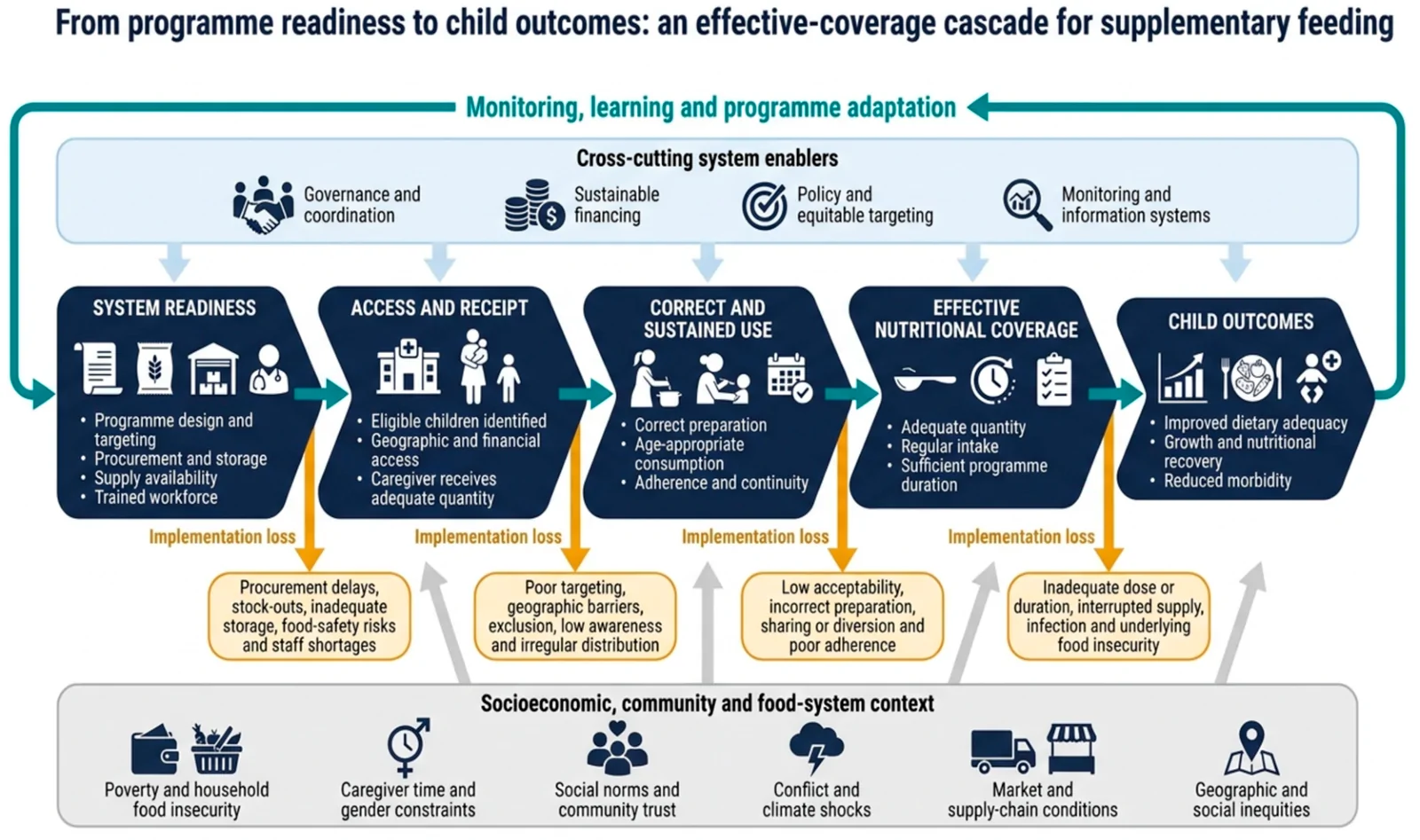
Multilevel implementation pathway linking supplementary feeding program delivery to effective coverage and child outcomes. Created in BioRender. Patni, M.A. (2026) https://BioRender.com/dp7mrv9. Arrows represent conceptual links rather than empirically established causal effects.

These implementation mechanisms operate along a sequential pathway extending from program design and procurement to frontline delivery, household utilization, child intake and nutritional outcomes. The pathway and its major potential failure points are presented in Figure 1.

## 4. Health-System and Structural Determinants

### 4.1. Procurement, Supply Chains and Financing

Reliable procurement and distribution are prerequisites for continuity. Studies of acute malnutrition and supplementary feeding systems describe transport bottlenecks, inadequate buffer stock, weak storage, shortages of trained staff and inconsistent availability of nutrition commodities [7,11,12,13,14]. Geographic distance amplifies these problems in rural and pastoral settings. Local production may reduce import dependence and improve cultural familiarity, but only when inputs, food safety, quality assurance and financing are dependable [13,19].

Donor dependence can produce rapid initial scale-up while leaving programs vulnerable to changes in external priorities. Evidence from Bangladesh described minimal government budget allocation for integrated acute malnutrition services, while multi-country scale-up studies identify subnational budget constraints affecting training, transport, supervision and monitoring [12,15]. Financing should therefore be assessed as a delivery function rather than only as a commodity budget.

### 4.2. Information Systems, Governance and Coordination

Nutrition information systems frequently face unstandardized indicators, incomplete reporting, limited analysis capacity and low user confidence. Bangladesh stakeholders described poor usability and trust when definitions and reporting practices differed across settings [20]. Multi-country evidence similarly identifies weak health-management information systems as a constraint on scaling maternal, child health and nutrition interventions [15].

Fragmented leadership can separate policy commitments from operational delivery. Integration requires defined responsibilities, referral pathways, budgets and feedback mechanisms across health, agriculture, social protection and water and sanitation sectors [12,14,21]. Without these arrangements, multisector language may coexist with parallel programs and duplicated reporting.

## 5. Workforce Development and Implementation Capacity

### 5.1. Training, Workload and Role Clarity

Community health workers and other frontline cadres commonly support screening, counselling, distribution, referral, follow-up and data collection. Their performance depends on preparation, manageable caseloads, supplies, role clarity and referral support [22,23,24,25]. In India, advice delivered through scaled home-visit programs was associated with more timely complementary feeding, greater feeding frequency and improved dietary diversity, illustrating the potential value of repeated household contact while not establishing the effectiveness of every CHW delivery model [23].

Indirect training evidence suggests that one-off instruction is insufficient when programs require complex assessment, counselling and monitoring tasks. Refresher training, supervised practice and health-literacy skills are recurrent enabling mechanisms [22,25,26]. These mechanisms are transferable but should not be presented as direct proof of supplementary feeding effectiveness.

### 5.2. Supportive Supervision, Motivation and Digital Tools

Weak supervision can reduce service quality and contribute to worker demotivation. Studies of undernourished children and adjacent community-health programs describe heavy workloads, limited equipment, unclear follow-up responsibilities and inadequate incentives [24,27,28]. Sustainable workforce strategies should combine training with supportive supervision, supplies, transport, recognition and realistic workload allocation.

Digital tools may facilitate communication, data capture and supervision, but direct evidence from supplementary feeding programs is limited. WhatsApp supervision groups in Kenya were feasible for information sharing and quality support, while broader digital-health evidence highlights connectivity, device access, digital literacy and maintenance costs [29,30]. Immunization and responsive-parenting studies suggest that reminders and hybrid delivery may improve contact with families [31,32]; they do not establish effectiveness in supplementary feeding. Digital approaches should therefore be piloted and evaluated for workload, access, uptake and child-level utilization before scale-up.

## 6. Household, Family and Community Determinants

### 6.1. Caregiver Understanding, Cultural Fit and Adherence

Caregiver knowledge interacts with household resources, autonomy, local food systems and service availability. Qualitative evidence from tribal communities in India shows that information alone may be insufficient when livelihoods, infrastructure and household decision-making constrain recommended practices [33]. A hospital-based study from South Gujarat provides only a local illustration of feeding-practice gaps and should not be interpreted as a national estimate [34].

Food acceptability is both sensory and social. Caregivers in a Dhaka informal settlement rated locally available foods more favorably than some novel supplementary products, suggesting that cultural familiarity can support use when nutritional adequacy and safety are maintained [35]. Conversely, intra-household sharing may reduce the quantity received by the target child, as observed in process evaluations of food baskets and other nutrition programs [11,36].

### 6.2. Family Engagement and Care Groups

Many early-childhood interventions remain oriented primarily toward mothers even when fathers, elders or other household members influence resources and feeding decisions. Father-inclusive and male-engagement studies identify possible benefits for nutrition knowledge and shared planning [37,38]; however, this evidence does not establish the effectiveness of family-engagement strategies within supplementary feeding programs. Breastfeeding evidence from Turkey further illustrates that extended-family involvement may support or constrain recommended practices depending on context; it remains indirect and should not be generalized to supplementary feeding [39].

Care groups use community-selected volunteers and peer support to reinforce practices and mobilize households. The protocol describing this evidence base should be used only to define the approach [40]; findings about motivation and sustainability should be supported by the completed realist synthesis, which identified organizational resources, group dynamics, community support and volunteer motivation as interacting mechanisms [41].

## 7. Program Design, Delivery and Equity

### 7.1. Distribution Continuity and Food Quality

Untimely distribution, interrupted schedules and declining food quality can reduce participation and trust. A small qualitative case study from Indonesia reported operational improvements after dedicated distribution teams, centralized processing and monitoring were introduced, but the design does not support attributing the reported percentage changes solely to those actions [7]. Indian program reviews similarly identify delayed financing, uneven monitoring and geographic barriers [42].

Program menus and commodities must balance nutrient specifications, cultural acceptability, cost, shelf stability and operational feasibility [8,19,35,43,44]. Standardization can support quality assurance, while local adaptation can improve acceptability and resilience. Neither approach is inherently superior; the appropriate balance depends on regulatory capacity, production scale, procurement reliability and local dietary patterns.

### 7.2. Targeting, Access and Integration

Targeted programs can concentrate resources on children with greatest need but require reliable identification, disaggregated data and active outreach. Passive enrollment can miss families who do not know the criteria, cannot reach distribution points or fear stigma. Blanket approaches may improve reach during widespread food insecurity but increase cost and potential leakage. Programs should therefore report coverage among eligible children, not only the number of rations distributed.

Integration with primary health care, sanitation and social-protection services may address multiple determinants and simplify referral; however, it can also increase workload and dilute technical focus when responsibilities and resources are unclear [14,21,45]. Integration should be evaluated by function—such as shared screening, referral, data or supervision—rather than treated as an all-or-none program characteristic.

## 8. Monitoring, Evaluation and Adaptive Management

Monitoring systems should connect inputs and activities with implementation outcomes such as availability, coverage, continuity, fidelity, acceptability and effective child consumption. Growth monitoring may support identification and referral, but a Cochrane review found uncertain effects on several child-health outcomes because interventions and measurements were heterogeneous [46]. Anthropometric outcomes alone cannot diagnose where the delivery pathway failed.

Data collection has limited value when frontline staff do not receive feedback or when indicators are duplicated across vertical systems. Nutrition-information studies identify the need for standardized definitions, data-quality checks, decentralized analysis and visible corrective action [20,47]. A focused indicator set may improve completeness and reduce reporting burden, but this proposition should be tested against decision usefulness rather than assumed.

The multilevel implementation determinants identified across the evidence, together with their supporting evidence and likely program consequences, are summarized in Table 2.

**Figure 2:**
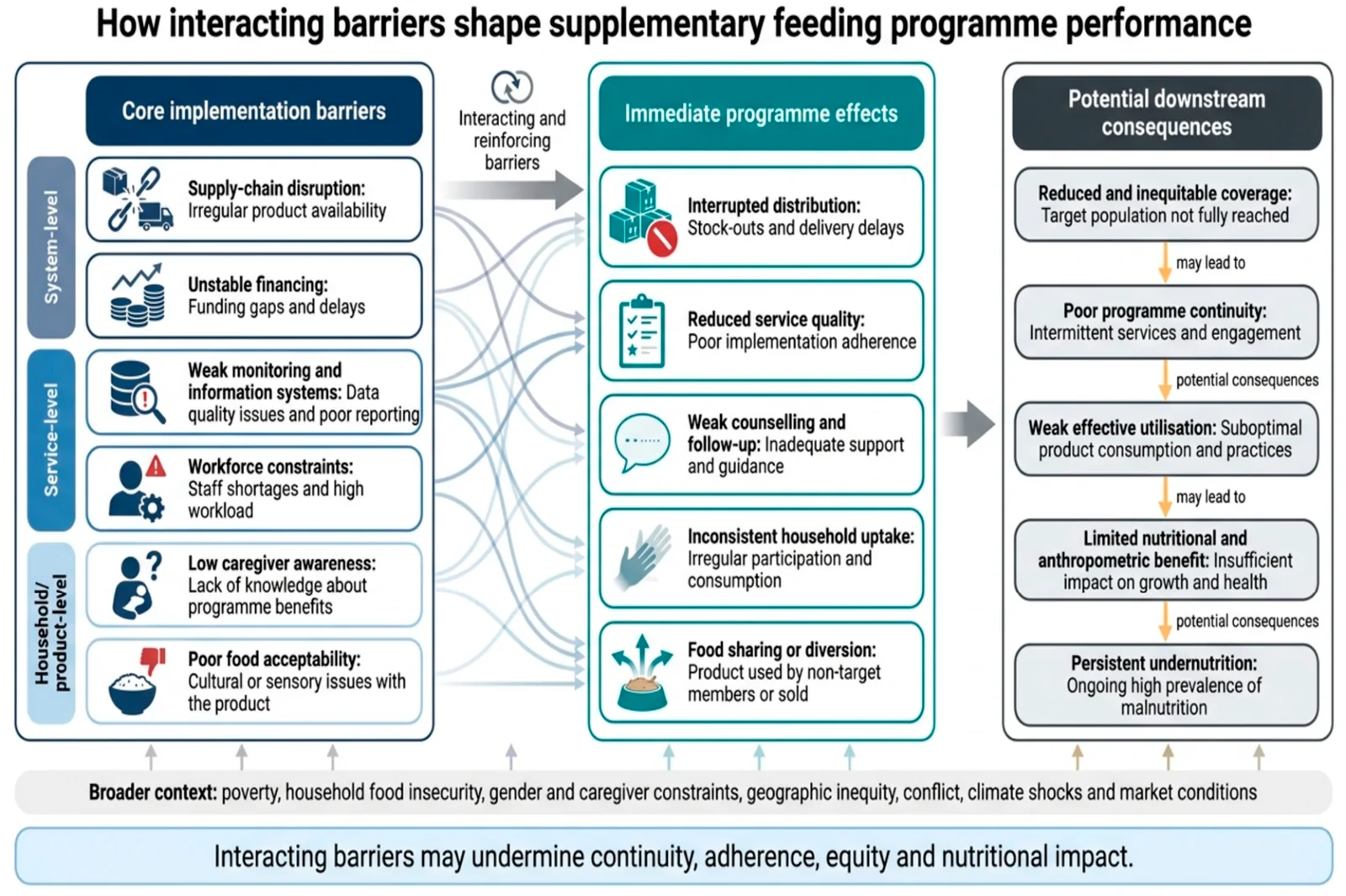
Conceptual pathways linking interacting implementation barriers to supplementary feeding program performance and downstream nutritional consequences. Created in BioRender. Patni, M.A. (2026) https://BioRender.com/4b9cf14. Arrows represent conceptual links rather than empirically established causal effects.

Across these domains, implementation barriers may interact and reinforce one another, producing cumulative effects on distribution continuity, service quality, household uptake, effective child consumption and nutritional outcomes (Figure 2).

## 9. Implementation Strategies and Major Trade-Offs

### 9.1. National Ownership Versus Donor-Supported Acceleration

External financing and technical support can accelerate introduction, but sustained coverage requires institutionalization within government budgets, training systems, procurement and information platforms. Evidence from zinc scale-up and donor-transition studies is indirect but consistently warns that coverage may decline when domestic systems are not strengthened before donor exit [49,55]. Broader scale-up evidence similarly emphasizes political commitment, adaptability, local capacity and long-term financing [50].

### 9.2. Centralized Standardization Versus Local Production

Centralized production can simplify product specifications and quality control, whereas local production may reduce transport costs and improve cultural familiarity. Local production is not automatically cheaper or safer: it requires predictable ingredients, testing, regulatory oversight, storage and skilled staff [13,19]. Hybrid models may be appropriate, with central standards and decentralized production or menu adaptation under verified quality systems.

### 9.3. Vertical Fidelity Versus Service Integration

Vertical programs may protect technical fidelity and commodity accountability, while integrated delivery may improve reach and continuity. Integration can nevertheless overload primary-care workers or obscure program responsibility. The decision should be based on which functions can be shared without reducing quality, supported by staffing, financing and governance arrangements [12,14,21].

### 9.4. Digital Efficiency Versus Workload and Exclusion

Digital reporting and messaging may shorten feedback cycles, but poorly designed systems can duplicate documentation and exclude workers or families with limited connectivity. Because evidence is primarily indirect, evaluations should compare digital, hybrid and non-digital delivery routes and measure total workload, data use, coverage and equity before any broad implementation recommendation is made [29,30,31,32].

## 10. Discussion

### 10.1. Principal Findings and Conceptual Contribution

This review indicates that supplementary feeding performance is produced by an interconnected delivery pathway rather than by food provision alone. Financing and governance shape procurement; procurement determines availability; workforce capacity influences counselling and follow-up; household resources and acceptability determine effective child intake; and monitoring determines whether programs adapt when these links fail. The central conceptual contribution is therefore a distinction between commodity availability and effective utilization: food can be procured and distributed without reaching the intended child in adequate quantity, frequency or duration.

The pathway presented in Figure 1 and Figure 2 is an author-developed conceptual synthesis derived from recurring patterns across the included evidence. It organizes plausible relationships and potential points of implementation loss but does not establish causal relationships, temporal ordering or effect magnitude.

This pathway complements established frameworks rather than replacing them. The Consolidated Framework for Implementation Research organizes determinants across the innovation, outer setting, inner setting, individuals and implementation process [56]. RE-AIM emphasizes reach, effectiveness, adoption, implementation and maintenance [57], while the WHO health-system building blocks organize service delivery, workforce, information, medical products, financing and governance [58]. The present review adds a nutrition-program-specific sequence that follows a product and service from policy and financing through procurement, frontline delivery, household receipt and actual child consumption. It also separates evidence directness from credibility at each stage. This makes it possible to locate where effective coverage is lost and to identify which proposed solutions are supported by supplementary feeding evidence and which remain transferable hypotheses.

The evidence also shows that barriers should not be ranked universally. A supply constraint may dominate during conflict or import disruption, while household sharing may be more important where food insecurity is severe and commodities are consistently available. Implementation assessment should identify the weakest link in a specific pathway and examine interactions rather than applying a uniform barrier checklist.

A child well-being perspective broadens the interpretation of program performance beyond anthropometry or ration delivery alone. Supplementary feeding can address an immediate dietary gap, but its wider contribution depends on whether it connects families to counselling, health care, social protection and supportive food environments. The child food poverty literature therefore reinforces the pathway model developed in this review: effective utilization is shaped not only by commodity availability but also by household resources, care, service access and local food systems [1,2,3,4]. Because the available well-being and household studies did not evaluate supplementary feeding interventions directly, these links should be treated as a conceptual rationale and an agenda for measurement rather than as evidence of broad well-being effects.

### 10.2. Directness, Geography and Interpretation

The strongest conclusions concern supply continuity, frontline-worker support, food acceptability, active outreach, usable monitoring and sustained financing, which were supported by direct or closely related evidence. However, direct evidence was concentrated in South Asia and selected programs in Iran and Ethiopia [36,51], limiting transferability across LMICs. Evidence concerning digital delivery, family engagement, specific community-health-worker incentive models and donor transition was mainly indirect and should therefore be treated as hypothesis-generating rather than as proof of effectiveness in supplementary feeding programs.

### 10.3. Implications for Policy and Practice

Program plans should specify the full pathway from eligibility and procurement to child consumption and outcome monitoring. Budgets should account for supervision, transport, storage, quality assurance, data use and community engagement. Coverage indicators should be disaggregated by geography and vulnerability, and process indicators should identify whether discontinuity occurred at supply, service, household or consumption stages. These are diagnostic priorities rather than a universal implementation package; the appropriate response must be selected after assessing the local delivery platform, administrative capacity, food system and excluded populations.

A practical improvement cycle may combine routine indicator review with qualitative feedback from caregivers and frontline workers, but the effect of this approach should be evaluated. Figure 1 presents the delivery pathway, Figure 2 summarizes interacting barriers, Table 1 distinguishes the contribution of direct, related and indirect evidence, and Table 2 maps recurrent determinants to likely consequences and supporting evidence.

### 10.4. Strengths and Limitations

The review’s strengths include its multilevel scope, clear distinction between evidence-directness categories, integration of empirical and operational evidence and focus on implementation mechanisms rather than commodity efficacy alone. The supplementary evidence matrix improves transparency by presenting the design, setting, relevance and limitations of each source.

Important limitations remain. The original searches were iterative and not prospectively archived; therefore, exact historical search strings, exhaustive retrieval and record-level flow counts cannot be claimed. This partial reproducibility may have influenced the composition and thematic balance of the included evidence and prevents full assessment of selection bias. Selection and charting were not conducted through duplicate independent procedures, and the English-language restriction may have introduced language bias. No formal design-specific risk-of-bias assessment was undertaken. The author-developed narrative credibility categories were used solely as an interpretive aid and should not be regarded as substitutes for validated critical-appraisal, risk-of-bias or certainty-assessment methods. Possible overlap between primary studies and reviews, publication bias, geographic concentration, heterogeneous programs and outcomes and reliance on indirect evidence further limit causal interpretation and transferability. Consequently, this synthesis cannot rank implementation barriers universally or establish the effectiveness of proposed strategies across all LMIC settings.

### 10.5. Research Priorities

Future research should prioritize four areas. First, prospective studies should test whether improvements in supply continuity, supervision, monitoring feedback and culturally acceptable delivery improve effective child-level consumption. Second, hypotheses derived mainly from indirect evidence, including digital reminders, father-inclusive delivery, community-health-worker incentives and donor-transition strategies, require evaluation within supplementary feeding programs. Third, direct evidence is needed from fragile and conflict-affected settings, Latin America, the Middle East and under-represented African regions. Fourth, studies should use standardized implementation outcomes and report cost, equity, food sharing, adherence and sustainability. Hybrid effectiveness-implementation studies, realist evaluations and comparative process evaluations can help determine which strategies work, for whom and under what conditions.

## 11. Conclusions

The most consistent direct and related evidence indicates that supplementary feeding programs depend on reliable financing and supply systems, prepared and supported frontline workers, acceptable foods, active outreach, equitable access and monitoring linked to corrective action. Programs should measure whether the intended child receives and consumes the food in the required quantity, frequency and duration, because distribution alone does not establish effective utilization.

The geographic breadth and methodological strength of direct implementation evidence remain limited. Digital reminders, father-inclusive delivery, specific community-health-worker incentive models and donor-transition strategies are supported mainly by related or indirect evidence and should be treated as hypotheses requiring prospective evaluation. Context-adapted implementation is warranted, but the present synthesis cannot identify a universally preferred delivery model across LMICs.

## Figures and Tables

**Table 1:** Contribution of direct, related and indirect evidence to the main conclusions.

Main Conclusion	Direct and Related Evidence	Indirect Evidence Contribution	Interpretation
Supply continuity and product availability	Direct program evaluations and related nutrition services repeatedly identified procurement delays, stock-outs, transport and storage constraints.	Donor-transition and health-system studies explain sustainability risks.	One of the best-supported operational conclusions, although the preferred supply model remains context-specific.
Workforce preparation and supervision	Direct and related studies identified training gaps, workload, role clarity and weak follow-up.	Broader community-health-worker evidence informs possible supervision and incentive mechanisms.	Workforce support is supported; specific incentive or supervision models require direct testing.
Acceptability and child-level utilization	Direct and related studies support attention to food acceptability, caregiver understanding and intra-household sharing.	Broader family and feeding studies explain household decision pathways.	Programs should measure actual child consumption; the best engagement strategy will vary by setting.
Monitoring linked to corrective action	Nutrition-program evidence identifies incomplete, duplicative and poorly used routine data.	Health-information and growth-monitoring evidence informs indicator design and feedback mechanisms.	The need for usable monitoring is supported; optimal indicator sets have not been established.
Digital delivery and family engagement	Direct supplementary feeding evidence is limited.	Digital-health, immunization, parenting and father-inclusive studies suggest potentially transferable mechanisms.	These are implementation hypotheses and should not be presented as proven supplementary feeding strategies.
National ownership and long-term sustainability	Direct evidence on post-donor transition in supplementary feeding is limited.	Donor-transition, scale-up and adjacent nutrition evidence indicates possible financing and institutionalization risks.	The direction of concern is plausible, but specific transition strategies require prospective evaluation.

**Table 2:** Multilevel implementation determinants, supporting evidence and likely program consequences. Consequences represent the authors’ synthesis and should not be interpreted as pooled causal effects.

Level	Recurrent Determinants	Supporting References	Likely Implementation Consequence
Policy and governance	Fragmented leadership, weak multisector coordination, donor dependence and unclear operational responsibility	[12,14,15,21,42,48,49,50]	Uneven implementation, duplication, weak accountability and poor sustainability
Financing, procurement and logistics	Insufficient domestic budgets, procurement delays, transport and storage constraints, inadequate buffer stock	[7,11,12,13,14,19,44,51]	Stock-outs, interrupted distribution, declining trust and reduced continuity
Workforce and supervision	Inadequate preparation, excessive workload, turnover, weak supportive supervision and limited incentives	[11,12,23,24,25,26,27,28,29,51]	Variable counselling quality, weak follow-up and reduced implementation fidelity
Monitoring and information systems	Unstandardized indicators, incomplete data, limited analysis capacity and duplicative reporting	[15,20,46,47]	Delayed corrective action, weak equity monitoring and reporting fatigue
Program design and delivery	Poor targeting, untimely schedules, limited cultural acceptability and weak integration with other services	[7,8,19,21,35,36,42,43,44,45,52]	Reduced reach, food wastage, diversion and incomplete exposure
Household and family	Limited caregiver understanding, competing priorities, food sharing, constrained autonomy and uneven family support	[7,33,34,35,36,37,38,39,41,53,54]	Low adherence, reduced child-level intake and irregular attendance
Community, access and equity	Long travel distances, weak mobilization, poverty, food insecurity and exclusion of remote or marginalized groups	[11,22,24,33,41,48,51]	Persistent inequity, low coverage and weak local ownership

## Data Availability

No new data were created or analyzed in this study. Data sharing is not applicable to this article.

## Supplementary Materials

The following supporting information can be downloaded at: https://www.mdpi.com/article/10.3390/children13101392/s1, Supplementary Box S1: Search documentation and reproducible verification strategy; Supplementary Table S1: Source-level evidence chart for the narrative synthesis.

## Abbreviations

The following abbreviations are used in this manuscript:

**Abbreviation**	**Full Form**
CHW/CHWs	Community Health Worker/Community Health Workers
CMAM	Community-Based Management of Acute Malnutrition
LMIC/LMICs	Low- and Middle-Income Country/Low- and Middle-Income Countries
MEDLINE	Medical Literature Analysis and Retrieval System Online
PRISMA	Preferred Reporting Items for Systematic Reviews and Meta-Analyses
PRISMA-S	Preferred Reporting Items for Systematic Reviews and Meta-Analyses Literature Search Extension
RAK	Ras Al Khaimah
SANRA	Scale for the Assessment of Narrative Review Articles
UNICEF	United Nations Children’s Fund
WHO	World Health Organization
