# Supplementary material for: Implementation Determinants and System-Level Barriers in Supplementary Feeding Programs for Children Under Five in Low- and Middle-Income Countries: A Critical Narrative Review
Source: Children (Basel). 2026 Oct 13;13(10):1392. doi: 10.3390/children13101392 (PMC13703596; doi:10.3390/children13101392)
Supplement: Supplementary file 1 [file children-13-01392-s001.zip › children-4563954-supplementary.pdf]

SUPPLEMENTARY MATERIAL

Supplementary Box S1. Search documentation and reproducible verification strategy

The discovery searches in PubMed/MEDLINE, Scopus, Web of Science Core Collection, Google Scholar and the Cochrane Library were iterative. Exact historical strings, search dates and record-level exports were not prospectively archived and cannot be reconstructed without introducing false precision. The strategy below documents the reproducible PubMed query used for final targeted verification on 18 August 2026. Equivalent concept blocks informed the earlier database searches, but they are not presented as exact historical logs.

PubMed/MEDLINE final verification query

```
(
  infant*[Title/Abstract] OR child*[Title/Abstract] OR toddler*[Title/Abstract]
  OR preschool*[Title/Abstract] OR under-five[Title/Abstract]
  OR "under five"[Title/Abstract] OR "0-59 months"[Title/Abstract]
  OR "6-59 months"[Title/Abstract]
)
AND
(
  "supplementary feeding"[Title/Abstract] OR "supplemental feeding"[Title/Abstract]
  OR "supplementary nutrition"[Title/Abstract] OR "take-home ration*"[Title/Abstract]
  OR "hot cooked meal*"[Title/Abstract] OR "fortified blended food*"[Title/Abstract]
  OR "ready-to-use supplementary food*"[Title/Abstract] OR RUSF[Title/Abstract]
  OR "ready-to-use therapeutic food*"[Title/Abstract] OR RUTF[Title/Abstract]
  OR "lipid-based nutrient supplement*"[Title/Abstract]
  OR "community-based management of acute malnutrition"[Title/Abstract] OR CMAM[Title/Abstract]
)
AND
(
  implement*[Title/Abstract] OR barrier*[Title/Abstract] OR facilitator*[Title/Abstract]
  OR deliver*[Title/Abstract] OR acceptab*[Title/Abstract] OR adheren*[Title/Abstract]
  OR uptake[Title/Abstract] OR participation[Title/Abstract] OR coverage[Title/Abstract]
  OR access*[Title/Abstract] OR "supply chain*"[Title/Abstract] OR procurement[Title/Abstract]
  OR distribution[Title/Abstract] OR workforce[Title/Abstract]
  OR "community health worker*"[Title/Abstract] OR supervision[Title/Abstract]
  OR monitor*[Title/Abstract] OR financing[Title/Abstract] OR governance[Title/Abstract]
  OR coordination[Title/Abstract] OR equity[Title/Abstract] OR sustainab*[Title/Abstract]
  OR "scale-up"[Title/Abstract] OR integration[Title/Abstract]
)
AND
(
  "Developing Countries"[MeSH] OR "low-income countr*"[Title/Abstract]
  OR "middle-income countr*"[Title/Abstract]
  OR "low- and middle-income countr*"[Title/Abstract] OR LMIC*[Title/Abstract]
  OR Africa[Title/Abstract] OR Asia[Title/Abstract] OR "Latin America"[Title/Abstract]
)
```

Organizational-source verification. Targeted searches of WHO, UNICEF, World Food Programme and relevant government websites combined the terms supplementary feeding, supplementary nutrition, take-home ration, ready-to-use supplementary food, CMAM, implementation, guidance, evaluation, monitoring, supply, workforce and sustainability. Citation chaining and searches for named programs were used to verify operational reports and current guidance. These targeted website searches were intended to verify key sources rather than provide exhaustive grey-literature coverage.

Contextual literature update. On 29 September 2026, targeted searches combined child food poverty, nutrition poverty, dietary deprivation and child well-being with terms for children under five and LMICs. These sources were used to frame the broader problem and its determinants, not to establish the effectiveness of supplementary feeding programs.

Supplementary Table S1. Source-level evidence chart for the narrative synthesis

Interpretive note. Direct evidence evaluated a supplementary-feeding or supplementary-nutrition programme for children under five. Related evidence concerned CMAM, therapeutic feeding, complementary feeding or broader child-nutrition services with aligned delivery mechanisms. Indirect evidence came from adjacent delivery fields and was used to explain plausible mechanisms rather than establish supplementary-feeding effectiveness. Narrative credibility was judged from clarity of the objective and setting, appropriateness of design, transparency of sampling and analysis, acknowledgement of limitations, and consistency between evidence and interpretation. Higher credibility denotes methodologically explicit guidelines or evidence syntheses; moderate credibility denotes peer-reviewed empirical or process-evaluation studies with methods sufficient for interpretation; contextual credibility denotes protocols, narrative reviews, selected grey literature or limited/remote evidence. These categories are author-developed, are not validated risk-of-bias ratings and do not establish certainty. Direct or related evidence of higher or moderate credibility was given the greatest weight in the synthesis. Indirect or contextual evidence generated hypotheses and did not independently justify recommendations. Sources 1, 2 and 4 provide contextual framing for child food poverty and child well-being, while Source 3 provides indirect LMIC household evidence; none directly evaluates a supplementary feeding program.

Citation	Country/region	Design/source type	Population or programme	Implementation determinant(s)	Reported or likely implementation consequence	Evidence directness	Principal limitation	Narrative credibility
**1. UNICEF (2024)**	Global, with emphasis on LMICs	Global report and cross-country descriptive analysis	Children in early childhood, including children under five living in child food poverty	Diet diversity, food environments, feeding practices, household income poverty, health services, social protection and data systems	Child food poverty threatens survival, growth and development; coordinated system action may improve access to nutritious diets	Indirect (contextual)	Global descriptive report; not a supplementary-feeding implementation evaluation	Higher (contextual)
**2. Leriou (2023)**	Attica, Greece	Cross-sectional survey and multidimensional indicator analysis	School-age children participating in Round Five of the C.W.-SMILE study	Nutrition, home conditions, guardian unemployment, health-care access, moral education and leisure	Demonstrates that nutrition contributes to a broader multidimensional construct of child well-being	Indirect (conceptual)	High-income setting and school-age population; not an under-five supplementary-feeding program	Moderate
**3. Otekunrin and Otekunrin (2022)**	Nigeria	Cross-sectional farm-household study	Under-five children in smallholder farm households	Household commercialization, economic conditions and nutritional status	Shows that higher commercialization did not consistently correspond to better child nutritional status	Indirect	Not a supplementary-feeding program; small child subsample and cross-sectional design	Moderate
**4. Prasad and Rohloff (2024)**	Global	Peer-reviewed commentary	Children experiencing food poverty	Diet quality, structural inequity, food environments, commercial influences and social policy	Supports system-level responses that address food quality and structural determinants rather than hunger alone	Indirect (contextual)	Commentary without primary data or supplementary-feeding evaluation	Contextual
**5. WHO (2023)**	Global	Evidence-informed guideline	Infants and children <5 years with wasting or nutritional oedema	Eligibility, assessment, treatment pathway, referral and follow-up	More consistent case management and service delivery	Related	Normative guidance; not an implementation evaluation	Higher
**6. Keats et al. (2021)**	LMICs	Evidence review/update	Maternal and child nutrition interventions	Intervention package, delivery platform, coverage and context	Potential gains in nutrition and survival when coverage is adequate	Related	Broad efficacy synthesis with limited implementation detail	Higher
**7. Nasution et al. (2025)**	Rural Indonesia	Qualitative case study	Supplementary feeding programme for malnourished toddlers	Distribution, processing, monitoring, staffing and access	Participation and continuity may improve when operational bottlenecks are corrected	Direct	Small local case; reported changes cannot establish causality	Moderate
**8. Ayoob et al. (2024)**	India	Optimization modelling	Diets for a child supplementary nutrition programme	Nutrient specifications, ingredient availability and cost	Feasible lower-cost formulations under stated constraints	Direct	Model assumptions and prices; no field implementation outcomes	Moderate
**9. Vazir et al. (2013)**	Rural India	Cluster-randomized trial	Toddlers and caregivers receiving complementary/responsive feeding education	Repeated caregiver counselling and responsive feeding support	Improved intake and modest growth/development gains	Related	Education-focused intervention; transfer to supplementary feeding is uncertain	Moderate
**10. Panjwani & Heidkamp (2017)**	LMICs	Systematic review and meta-analysis	Complementary feeding interventions in young children	Intervention content, intensity, age and baseline nutritional status	Small average gains in linear and ponderal growth	Related	Substantial heterogeneity; limited program-process evidence	Higher
**11. Beggs et al. (2024)**	Low-/lower-middle-income countries	Scoping review	Complex community interventions addressing acute malnutrition	Supply, workforce, community engagement, access and coordination	Reach, fidelity and sustainability depend on interacting system conditions	Related	Diverse designs and programmes; no pooled effects	Higher
**12. Ireen et al. (2018)**	Bangladesh	Qualitative study	CMAM stakeholders and government health-system actors	Governance, financing, workforce, supplies, monitoring and integration	Fragmented or incomplete integration can weaken continuity and scale	Related	Stakeholder perceptions from one country	Moderate
**13. Segre et al. (2017)**	Global/UNICEF supply market	Operational and economic analysis	RUTF and SQ-LNS manufacturers and procurement systems	Local vs offshore production, taxation, capacity use, finance, inputs and testing	Cost, supply reliability and producer sustainability vary by production model	Related	Historical procurement data; no child or programme-effect outcomes	Moderate

**14. Kouam et al. (2014)**	Bangladesh	Qualitative study	CMAM implementers and local health-system stakeholders	Roles, financing, referral pathways, workforce and local ownership	Clearer integration arrangements may improve continuity and accountability	Related	Single-country stakeholder evidence	Moderate
**15. Coffey et al. (2024)**	Burkina Faso, Ethiopia, India, Kenya and Nigeria	Exploratory mixed-methods assessment	Subnational implementers of 14 MNCHN assets	Policy, financing, supply, training, demand generation and data use	System readiness shapes progress toward scale and effective coverage	Indirect	Perceived progress and non-uniform assets; not feeding-specific	Moderate
**16. Baethge et al. (2019)**	International	Methodological scale development/validation	Narrative review articles	Justification, objectives, search description, referencing and scientific reasoning	More transparent assessment of narrative-review reporting quality	Indirect (methodological)	Not programme evidence and not a formal risk-of-bias tool	Higher (methodological)
**17. Sukhera (2022)**	International	Methodological commentary	Authors and readers of narrative reviews	Explicit purpose, transparent searching and critical interpretation	Improved rigor and usability of narrative synthesis	Indirect (methodological)	Non-empirical guidance	Contextual
**18. Rethlefsen et al. (2021)**	International	Reporting-guideline extension	Literature searches for evidence syntheses	Database, strategy, limits and search-process reporting	Greater reproducibility and transparency of searches	Indirect (methodological)	Designed for systematic reviews; only partly transferable here	Higher (methodological)
**19. Fetriyuna et al. (2023)**	LMIC/local-resource settings	Technical narrative review	Locally produced RUTF/RUSF and related products	Technology access, ingredients, production, quality assurance and sustainability	Local products may improve access but require reliable quality systems	Direct	Heterogeneous product studies; mainly short-term and non-pooled evidence	Contextual
**20. Fesshaye et al. (2024)**	Bangladesh	Qualitative study	Users and stakeholders of the Nutrition Information System dashboard	Data quality, usability, definitions, trust and analytic capacity	Poor usability and trust can limit routine data use and corrective action	Related	Dashboard-focused, perception-based evidence	Moderate
**21. Perez-Escamilla & Engmann (2019)**	Global	Expert review/commentary	Nutrition services delivered through health-system platforms	Integration, governance, workforce, financing and platform readiness	Integration may improve reach but can dilute responsibility or overload services	Related	Broad conceptual synthesis; not supplementary-feeding specific	Contextual
**22. Ahmed et al. (2022)**	LMICs	Systematic review	Community health workers and populations facing inequity	Selection, remuneration, supervision, accountability and community embeddedness	CHW design can improve or reproduce inequities in access	Indirect	Heterogeneous CHW roles; little direct feeding evidence	Higher
**23. Garg et al. (2023)**	Rural Chhattisgarh, India	Cross-sectional household survey with multivariable analysis	2,646 households with children aged 7-36 months in scaled HBYC	CHW home visits, counselling coverage and repeated household contact	Advice was associated with better feeding practices and use of supplementary food	Related	Cross-sectional self-report; association does not prove causation	Moderate
**24. Njeru et al. (2021)**	Bangladesh and Kenya	Qualitative stakeholder study	CHWs and stakeholders supporting ill, undernourished children after discharge	Role clarity, equipment, training, referral, follow-up and incentives	Weak support may interrupt recovery follow-up and continuity	Related	Post-discharge context; stakeholder perceptions	Moderate
**25. O'Donovan et al. (2018)**	LMICs	Systematic scoping review	Ongoing training programmes for CHWs	Training frequency, modality, supervision and organizational support	Continuing support may improve competence and service quality	Indirect	Heterogeneous programmes and outcomes	Higher
**26. Fung et al. (2021)**	LMICs	Scoping review	Training to identify sick newborns and prompt facility care-seeking	Training content, reinforcement, assessment and referral support	Better recognition and referral may improve continuity of care	Indirect	Newborn-care evidence; remote from supplementary feeding	Higher
**27. Berhanu et al. (2025)**	Ethiopia	Qualitative pilot evaluation	Stakeholders implementing antenatal multiple micronutrient supplementation	Supply, training, counselling, workflow and acceptability	Operational readiness influences coverage and fidelity	Indirect	Maternal antenatal programme and pilot setting	Moderate
**28. Akinola et al. (2025)**	Zambia	Qualitative case study	Community health assistants delivering maternal primary care	Workload, role definition, training, incentives and supplies	Constraints may reduce service quality and follow-up	Indirect	Maternal-health context; limited transferability	Moderate
**29. Henry et al. (2016)**	Two low-resource Kenyan settings	Qualitative feasibility study	CHWs and supervisors using WhatsApp for supervision	Connectivity, moderation, device access and supervisory responsiveness	Faster communication and peer support, with risk of exclusion or added workload	Indirect	Small purposive samples; no nutrition or clinical outcome evaluation	Moderate
**30. Mishra et al. (2019)**	LMICs	Narrative review	CHWs using digital tools for NCD programmes	Connectivity, digital literacy, maintenance, workload and governance	Digital tools may support delivery but can create duplication and exclusion	Indirect	NCD-focused and non-systematic	Contextual
**31. Kazi et al. (2018)**	Pakistan	Randomized controlled trial	Caregivers receiving mobile reminders for routine immunization	SMS reminders and family contact	Improved service uptake in an adjacent delivery setting	Indirect	Different intervention and outcome; mechanism transfer only	Moderate

**32. Garcia et al. (2024)**	Rural Kenya	Cluster-randomized trial	Caregivers in a responsive-parenting intervention	Remote vs in-person delivery, access, engagement and fidelity	Hybrid delivery may maintain family contact under some conditions	Indirect	Parenting rather than feeding; context-specific delivery comparison	Moderate
**33. Lakhanpaul et al. (2022)**	Tribal communities, India	Qualitative analysis	Caregivers, families and community stakeholders involved in child feeding	Knowledge, livelihoods, infrastructure, norms and household decision-making	Information alone may not change feeding when structural constraints persist	Related	Small number of communities; context-specific	Moderate
**34. Choraria et al. (2023)**	South Gujarat, India	Facility-based cross-sectional study	Caregivers and children aged 0-24 months attending an outpatient department	Caregiver knowledge and infant/young-child feeding practices	Local feeding-practice gaps may reduce dietary adequacy	Related	Single facility, self-report and limited generalizability	Moderate
**35. Rahman et al. (2026)**	Dhaka informal settlement, Bangladesh	Formative Trials of Improved Practices study	Caregivers and moderately wasted children exposed to novel and local foods	Sensory/cultural acceptability, preparation and caregiver use	Acceptability shapes uptake and actual child consumption	Direct	Small, short formative study; not an effectiveness trial	Moderate
**36. Ghodsi et al. (2018)**	Iran	Cross-sectional process evaluation using observations, documents, interviews and focus groups	National community-based food supplementary programme for children	Targeting, food-basket composition, sharing, training and monitoring	Implementation gaps can reduce fidelity and child-level intake	Direct	Cross-sectional process data; no counterfactual	Moderate
**37. Jeong et al. (2023)**	LMICs	Systematic review	Father-inclusive early-childhood interventions	Recruitment, intensity, delivery mode and degree of father engagement	Family-inclusive design may strengthen participation and shared planning	Indirect	Few nutrition-specific interventions and heterogeneous outcomes	Higher
**38. Thakur et al. (2025)**	Bihar, India	Quasi-experimental baseline/endline evaluation with difference-in-differences	Mothers, fathers and children aged 6-23 months	Male engagement, couple home visits, behavioural prompts and community support	Improved dietary diversity, knowledge and joint food planning	Related	Non-random block selection and short follow-up	Moderate
**39. Celebier et al. (2025)**	Ankara, Turkiye	Facility-based cross-sectional survey	392 mothers of children aged 2-5 years	Family influence, socioeconomic conditions, delivery mode and perceived milk insufficiency	Cultural/family context may support or constrain feeding practices	Indirect	Retrospective maternal report from one hospital; breastfeeding-focused	Moderate
**40. Pieterse et al. (2020)**	LMICs	Realist review protocol	Care Groups promoting infant feeding	Proposed mechanisms, contexts and outcomes	Defines how the Care Group evidence was to be synthesized	Related	Protocol only; no completed findings	Contextual
**41. Pieterse et al. (2022)**	LMICs	Realist synthesis	Care Groups and community volunteers promoting social/behaviour change	Organizational resources, group dynamics, community support and volunteer motivation	Mechanisms influence volunteer continuity and household engagement	Related	Mechanism-focused evidence with heterogeneous underlying studies	Higher
**42. Rao & Kaul (2018)**	India	Programme/policy review	Integrated Child Development Services	Scale-up, governance, workforce preparation, quality and local adaptation	Rapid expansion without system support can produce uneven implementation	Direct	Secondary narrative synthesis across diverse states	Contextual
**43. Dhasmana et al. (2025)**	Andhra Pradesh, India	Qualitative study	ICDS supplementary nutrition stakeholders and programme menus	Dietary diversity, environmental sustainability, procurement and local food adaptation	Menu reform may improve acceptability and diversity if operationally feasible	Direct	One-state qualitative evidence; no effectiveness estimate	Moderate
**44. NITI Aayog & WFP (2022)**	India	Government/agency good-practice compendium	Take-home ration systems across states/union territories	Production, procurement, quality control, distribution and monitoring	Operational models may improve continuity and product quality	Direct	Grey literature; descriptive selection of practices without comparative evaluation	Contextual
**45. Singh et al. (2024)**	India	Cross-sectional secondary-data analysis	Children and families eligible for Integrated Child Development Services	Service access and utilization	Unequal utilization may limit population reach and nutrition benefit	Direct	Observational association with residual confounding and selection	Moderate
**46. Taylor et al. (2023)**	LMICs	Cochrane systematic review	Growth monitoring and promotion for children <5 years	Programme design, frequency, coverage, referral and counselling	Effects on several child-health outcomes were uncertain	Related	Heterogeneous interventions and generally low-certainty evidence	Higher
**47. Ouedraogo et al. (2025)**	Burkina Faso	Qualitative implementation study with document review, interviews and validation workshop	Nutrition routine data integrated into institutional HMIS/DHIS2	Indicator standardization, participation, capacity, data quality and feedback	Stronger systems can improve data availability and decision use	Related	Retrospective capitalization and implementer perspectives	Moderate

**48. Jalaludin et al. (2025)**	Nepal, Bangladesh and Vietnam	Literature review	Policies and programmes addressing stunting in children <5 years	Multisector integration, community delivery, monitoring and policy support	Coordinated implementation may improve reach and sustained nutrition action	Related	Narrative country review without pooled or causal estimates	Contextual
**49. Huffstetler et al. (2022)**	Middle-income countries	Scoping review	Health systems undergoing donor transition	Transition planning, domestic financing, procurement, workforce and institutional capacity	Poorly managed transition can reduce coverage and disrupt services	Indirect	Diverse health programmes; limited feeding-specific evidence	Higher
**50. Bulthuis et al. (2020)**	LMICs	Qualitative systematic literature review	Public-health interventions undergoing scale-up	Political commitment, leadership, financing, adaptability and local capacity	Scale and sustainability depend on system fit and long-term ownership	Indirect	Broad interventions and predominantly qualitative evidence	Higher
**51. Degefa et al. (2025)**	Ethiopia	Qualitative study	Supplementary feeding programmes for under-5 children with severe acute malnutrition	Supplies, workload, access, management, referral and delayed care-seeking	Constraints can interrupt coverage, continuity and adherence	Direct	Single setting; qualitative evidence cannot estimate prevalence or effect	Moderate
**52. Hussain & Bhutta (2026)**	Low-resource settings	Narrative review	Strategies to reduce childhood malnutrition	Prevention, integration, community delivery and food/nutrition support	Context-adapted packages may improve reach and outcomes	Related	Broad narrative review with limited implementation-specific appraisal	Contextual
**53. Sanghvi et al. (2023)**	Bangladesh, Burkina Faso, Ethiopia and India	Multi-country mixed-method intervention synthesis	Pregnant women and prenatal iron-folic acid programmes	Supply, counselling, training, side effects, community norms and data use	A comprehensive delivery approach can improve adherence	Indirect	Maternal supplementation; transferability to child feeding is indirect	Moderate
**54. Bairwa et al. (2025)**	Global/LMICs	Narrative review	Iron-folic acid supplementation among pregnant women	Awareness, side effects, supply, counselling and health-system support	Delivery barriers can reduce adherence and continuity	Indirect	Broad maternal-nutrition review; not child supplementary feeding	Contextual
**55. Beam et al. (2025)**	Bangladesh and other LMICs	Retrospective sustainability case study/review	Zinc treatment programmes for acute childhood diarrhoea	Policy, financing, procurement, training and demand after external support	Coverage may decline when domestic systems do not sustain scale-up	Indirect	Different intervention; retrospective attribution across settings	Moderate
**56. Damschroder et al. (2022)**	Multiple settings	Framework update based on user feedback	Implementation research across sectors	Innovation, outer setting, inner setting, individuals and implementation process	Provides an established determinant framework against which the review pathway can be compared	Indirect	General framework; not specific to child nutrition or supplementary feeding	Higher
**57. Glasgow et al. (1999)**	Multiple settings	Conceptual framework paper	Public-health intervention evaluation	Reach, effectiveness, adoption, implementation and maintenance	Provides evaluation dimensions relevant to program reach and sustainability	Indirect	General framework; not a supplementary-feeding evaluation	Higher
**58. WHO (2007)**	Global	Health-system framework	Health-system strengthening	Service delivery, workforce, information, products, financing, leadership and governance	Provides system domains relevant to structural determinants	Indirect	Normative framework; does not specify a supplementary-feeding delivery pathway	Higher

Abbreviations: CHW, community health worker; CMAM, community-based management of acute malnutrition; DHIS2, District Health Information Software 2; HBYC, Home-Based Care of Young Children; HMIS, health management information system; ICDS, Integrated Child Development Services; LMIC, low- and middle-income country; MNCHN, maternal, newborn, child health and nutrition; RUSF, ready-to-use supplementary food; RUTF, ready-to-use therapeutic food; SQ-LNS, small-quantity lipid-based nutrient supplements.
